# Passion Fruit Seed Oil as a Natural Tyrosinase Inhibitor: Extraction Optimization, Multi-Mechanism Elucidation, and Efficacy Validation in Zebrafish

**DOI:** 10.3390/foods15071246

**Published:** 2026-04-06

**Authors:** Jingyu Li, Zhihua Tao, Qingquan Guo, Yudong Zhang, Junhao Zhang, Yanlin Deng, Kegang Wu, Hongpeng Yu, Xianghua Chai, Yingfen Jiang, Dong He, Xiaoli Liu, Xuejuan Duan, Junfeng Liu

**Affiliations:** Department of Food Science and Engineering, Guangdong University of Technology, Guangzhou University Town 100, Guangzhou 510006, China; 18028282830@163.com (J.L.); qqg55555@163.com (Q.G.); 13322812398@163.com (Y.Z.); 18929029271@163.com (J.Z.); 13794382315@163.com (Y.D.); wukegang2003@163.com (K.W.); yuhpeng@163.com (H.Y.); xhchai813@sohu.com (X.C.); yingfen.jiang@gdut.edu.cn (Y.J.); hedong86284116@163.com (D.H.); jumper45@sina.com (X.L.); wandxjbb@163.com (X.D.); 18174847868@139.com (J.L.)

**Keywords:** passion fruit seed oil, supercritical carbon dioxide extraction, tyrosinase, zebrafish, downregulated genes

## Abstract

Tyrosinase promotes excessive deposition of melanin, which may lead to severe skin diseases. *Passiflora edulis f. edulis* seeds have been reported to be rich in diverse bioactive constituents exhibiting potential tyrosinase inhibitory activity. However, the principal bioactive constituents responsible for tyrosinase inhibitory activity and its underlying mechanisms remain largely unclear. Therefore, this study aimed to: (1) optimize SC-CO_2_ extraction of *Passiflora edulis f. edulis* seed oil (PFSO) for maximum yield and bioactive preservation; (2) comprehensively characterize its physicochemical and phytochemical profile; (3) elucidate the tyrosinase inhibition mechanism through kinetic, spectroscopic, and computational approaches; and (4) validate its safety, antioxidant, and anti-pigmentation efficacy in a zebrafish model. PFSO exhibited a yield of 24.96%, with a high content of unsaturated fatty acids (88.03%, mainly linoleic acid at 74.40%). The oil inhibited tyrosinase via a reversible mixed-type mechanism (IC_50_ = 1.12 mg/mL). Fluorescence spectroscopy and molecular docking revealed that linoleic acid binds to LYS180 and β-sitosterol binds to TYR78, mainly driven by hydrogen bonding and hydrophobic interaction, which changed the microenvironment of tryptophan residues and indicated static quenching. Further validation experiments revealed that the major constituent, linoleic acid, exhibited only weak inhibitory activity against tyrosinase (IC_50_ = 29.44 mg/mL), whereas the key component β-sitosterol markedly suppressed tyrosinase activity (IC_50_ = 46.43 μg/mL). In vitro assays demonstrated PFSO’s significant efficacy in reducing the melanin content and tyrosinase activity in α-MSH-stimulated B16F10 murine melanoma cells. In vivo experiments in zebrafish that received dietary supplementation with PFSO confirmed that PFSO (≤5 mg/mL) reduced ROS production, suppressed melanin deposition, inhibited tyrosinase activity, and downregulated the expression of melanogenesis-related genes (TYR, TYRP1, TYRP2, MITF). This study provides, for the first time, a comprehensive elucidation of PFSO’s potential as a natural tyrosinase inhibitor, integrating extraction optimization, multicomponent characterization, multimodal inhibition analysis, and in vivo validation.

## 1. Introduction

Melanin is a biogenic pigment produced by melanocytes residing in the basal layer of the epidermis [1]. Excessive accumulation of melanin may contribute to severe dermatological disorders [2]. Tyrosinase is the rate-limiting enzyme in melanin biosynthesis and a copper-containing metalloenzyme ubiquitously distributed across animals, plants, and microorganisms [3]. Inhibition of tyrosinase activity has long been a primary strategy for reducing melanin synthesis and achieving skin-whitening effects [4]. Conventional inhibitors (e.g., kojic acid, arbutin) face limitations such as skin irritation, instability, and regulatory restrictions [5]. Consequently, the development of safe, effective, and naturally derived tyrosinase inhibitors has become a major focus in dermatological and cosmetic research.

Plant-derived extracts have garnered attention as promising alternatives due to their multi-target activities and favorable safety profiles [6]. Passion fruit seeds (*Passiflora edulis*), part of the passion flower family, *Passifloraceae*, constitute up to 27% of fruit waste [7] and are rich in unsaturated fatty acids (notably linoleic acid), polyphenols, and tocopherols with a diverse array of biological activities, including antioxidant, antimicrobial, anti-inflammatory, antitumor, and hypolipidemic properties [8]. The lipid fraction of *Passiflora edulis* seeds is predominantly composed of unsaturated fatty acids, with linoleic acid (C18:2) being the principal component [9]. Preliminary studies suggest their tyrosinase inhibitory potential, yet their specific bioactive constituents, synergistic interactions, and underlying inhibitory mechanisms remain poorly elucidated.

The extraction method critically influences the bioactivity of plant oils. While conventional methods (such as pressing and Soxhlet extraction) may degrade thermolabile compounds [10] or leave solvent residues [11], supercritical CO_2_ extraction offers a green, low-temperature alternative that better preserves bioactive components [12]. However, the majority of investigations on PFSO have focused solely on extraction yield or basic compositional characterization; a systematic investigation linking extraction technology, chemical profile, and multi-level antimelanogenic activity is lacking. Although a few studies have reported tyrosinase inhibition by *Passiflora edulis* seed extracts [13], a comprehensive mechanism study—encompassing inhibition kinetics, conformational interaction, molecular docking, and in vivo validation—is still lacking. Furthermore, the role of specific fatty acids (e.g., linoleic acid) in tyrosinase inhibition, alongside the contribution of antioxidant phenolics, has not been deciphered.

Therefore, this study aimed to: (1) optimize SC-CO_2_ extraction of PFSO for maximum yield and bioactive preservation; (2) comprehensively characterize its physicochemical and phytochemical profile; (3) elucidate the tyrosinase inhibition mechanism through kinetic, spectroscopic, and computational approaches; and (4) validate its safety, antioxidant, and anti-pigmentation efficacy in a zebrafish model. Our work provides the first integrated, multi-mechanistic insight into the antimelanogenic action of PFSOs, establishing a scientific foundation for its application as a multifunctional natural ingredient in skin-whitening and antioxidative formulations.

## 2. Materials and Methods

### 2.1. Materials

*Passiflora edulis f. edulis* (PE 02114315) was identified and authenticated by Yingda Lin, Fengshan Laboratory, Taiwan. The voucher sample was kept at Fengshan Laboratory. Its seeds were purchased fromLINBobai Wanquan Fruit Industry Co., Ltd. (Yulin, China). The carbon dioxide cylinders used were purchased from Yuejia Gas Co., Ltd. (Guangzhou, China). Tocopherol, gallic acid, β-carotene, rutin, 2,2-Diphenyl-1-picrylhydrazyl (DPPH), Folin–Ciocalteu phenol reagent, α-melanocyte-stimulating hormone (α-MSH), L-DOPA, 2,2′-azino-bis(3-ethylbenzothiazoline)-6-sulphonic acid (ABTS), 2-Phenyl-4,4,5,5-tetramethylimidazoline-1-oxyl 3-Oxide (PTIO), linoleic acid, β-sitosterol, p-hydroxyacetophenone, 2’,7’-dichlorodihydrofluoresceine diacetate (H_2_DCFH-DA), anhydrous copper sulfate and tyrosinase (from mushrooms (*Agaricus bisporus*), ≥500 unit/mg solid) were purchased from Macklin Biochemical Technology. Co. (Shanghai, China). PBS (pH 6.8) was purchased from Haibiao Technology Co., Ltd. (Xiamen, China). The FRAP assay kit was sourced from Biosharp (Hefei, China). All other kits, including the Total RNA Column Extraction Kit, HiScript III RT SuperMix for qPCR, and ChamQ Universal SYBR qPCR Master Mix, were purchased from Vazyme (Nanjing, China). All other reagents were of analytical grade.

#### Animals

Wild-type AB strain zebrafish (*Danio rerio*) were acquired from the Chinese Zebrafish Resource Center (Wuhan, China). Zebrafish were reared in a recirculating system under a 14/10 h light–dark photoperiod at 28.5 ± 0.5 °C. The system water was maintained at a conductivity of 500–550 μS/cm and a pH of 7.0–8.0. Zebrafish were fed with newly hatched *Artemia nauplii* twice a day. One day prior to the experiment, healthy individuals were selected one hour after feeding and allocated to breeding tanks with males and females separated by a 1:1 ratio. The partition ended the next morning to initiate mating. Fertilized eggs were collected from the tank bottom after one hour, and healthy eggs were subsequently incubated at 28.5 ± 1 °C.

All zebrafish experiments were conducted in accordance with the OECD test guidelines (TG203 and TG236). All animal experimental procedures were approved by the Biomedical Research Ethics Committee of Guangdong University of Technology (protocol code 260120; date of approval: 26 March 2024).

### 2.2. Supercritical CO_2_ (SC-CO_2_) Extraction of PFSO

Following a previously published method with slight modifications [14], the dried and crushed *Passiflora edulis f. edulis* seeds were placed in a supercritical CO_2_ fluid extractor (Haoli Biotechnology Co., Ltd., Guangzhou, China). Single-factor experiments were employed to achieve the desired limits for each variable. All experiments were performed at least three times. The orthogonal array design was performed with minor modifications to a previously published method [15]. The effects of four factors—extraction temperature, pressure, static extraction time, and dynamic extraction time—on the extraction yield were evaluated using a three-level, four-factor orthogonal array design.

### 2.3. Analysis of Fatty Acid Composition of PFSO

Using the area normalization technique and the Chinese national standard (GB 5009.168-2016) as a guide, the fatty acid content of PFSO was determined [16]. Following methyl esterification, the fatty acid methyl ester composition of PFSO was measured using GC (Agilent 7890A). The chromatographic conditions were set as follows: column: DB-Fast FAME (100 m × 0.25 mm × 0.2 μm); carrier gas: helium; carrier gas flow rate: 3.0 mL/min; injection volume: 1.0 μL; initial temperature: 130 °C with a retention time of 5 min, rising to 220 °C at 4 °C/min with a retention time of 42 min; inlet temperature: 250 °C; and detector temperature: 250 °C. The relative fatty acid content was calculated using area normalization.

### 2.4. Identifying the Active Substance Content of PFSO

#### 2.4.1. Determination of Total Flavonoid Content (TFC)

Determination of TFC was measured using spectrophotometry, referring to previously published methods [17]. Briefly, 1 mL of PFSO was prepared using absolute ethanol as the solvent, and 1 mL of 5% NaNO_2_ was added. After that, 1 mL of 10% AlCl_3_ and 10 mL of 1% NaOH were added after 6 min. The absorbance value of mixtures was evaluated at 765 nm using a multifunctional microplate reader (SuperMax 3100, Flash, Shanghai, China). The standard curve was constructed based on the absorbance values of rutin standard solutions. The TFC was computed using the rutin standard curve, and a solution devoid of a sample served as the blank control.

#### 2.4.2. Determination of β-Carotene Content

β-carotene content determination was performed using a slightly modified version of Elik et al. [17]. After dissolving the β-carotene standard in n-hexane, it was fixed in 50 mL of methanol as a stock solution. After diluting the stock solution to 0.5–10 μg/mL with n-hexane to establish the standard curve, the absorbance was evaluated at 450 nm. In total, 1 g of PFSO was dissolved in n-hexane and diluted to 10 mL. After that, the absorbance was read at 450 nm, and its β-carotene content was established by a standard curve.

#### 2.4.3. Determination of Total Phenolic Content (TPC)

A previously published method was used to assess the TPC [18]. After dissolving gallic acid in methanol and diluting it to 10–50 μg/mL, 0.5 mL of the Folin–Phenol solution and 1.5 mL of a 75% Na_2_CO_3_ solution were added. Methanol was added to 10 mL and allowed to react in the dark for 2 h at room temperature. The standard curve was created by measuring the absorbance value at 765 nm. In total, 1 g of PFSO was mixed with 3 mL of methanol, sonicated for 5 min, and centrifuged to collect the supernatant (this procedure was repeated three times). The three supernatants were combined and added to 10 mL of methanol. A total of 0.1 mL of methanolic extract was transferred into a 10 mL volumetric flask, followed by the addition of 0.5 mL of Folin–Phenol solution to react for 3 min. After that, 1.5 mL of 75% Na_2_CO_3_ solution was added. Methanol was added to 10 mL and allowed to react in the dark for 2 h at room temperature. The absorbance value was evaluated at 765 nm. The gallic acid standard curve was utilized to quantify the total phenolic content using a solution devoid of a sample as the blank control.

#### 2.4.4. Determination of Vitamin E (Ve) Content

The Ve content was determined using the method outlined in the Chinese national standard (GB 5009.82-2016) [19]. In total, 1 g of PFSO was taken into a 25 mL volumetric flask, followed by the addition of 0.1 g of BHT. The sample was dissolved in 10 mL of mobile phase and diluted to 25 mL. The solution was filtered using a 0.22 μm organic membrane filter and determined using the HPLC system (Shimadzu LC2030). The following parameters were used: chromatographic column: Betasil Diol-100 (250 mm × 4.6 mm × 5 μm); injection volume: 10 μL; and mobile phase: n-hexane and 1,4-dioxane (95:5, *v*/*v*). The following parameters were used: isocratic elution: flow rate 0.8 mL/min; column temperature 30 °C; excitation detection wavelength: 294 nm; and emission wavelength: 328 nm. The concentrations of the target compounds in PFSO were computed based on the concentrations of the Ve standards, which were used to form the standard curve at concentrations ranging from 10 to 10–400 μg/mL.

### 2.5. Measurement of Radical Scavenging of PFSO

#### 2.5.1. Ferric-Reducing Antioxidant Power (FRAP) Assay

The FRAP assay was estimated according to previously published methods [20]. The FRAP solution was prepared according to the manufacturer’s instructions provided with the assay kit. In total, 100 μL of PFSO (1, 1.25, 2.5, 5, 10, and 20 mg/mL) at various concentrations was mixed with 20 μL of distilled water. The mixture was mixed with 680 μL of the FRAP solution and placed in the dark for 30 min at room temperature. The OD_590nm_ was evaluated. Trolox was used as a positive control, and results were expressed in μmol FeSO_4_/mL.

#### 2.5.2. DPPH Radical Scavenging Assay

The DPPH radical scavenging assay was evaluated according to previously published methods [21]. Briefly, 100 μL of PFSO (0.625, 1.25, 2.5, 5, 10, and 20 mg/mL) of various concentrations dissolved in absolute ethanol was dispensed into a 96-well plate and mixed with 100 μL of DPPH–absolute ethanol (0.2 mM). The mixture was incubated in the dark at room temperature for 30 min. OD_517nm_ was evaluated, and Trolox was employed as a positive control.

#### 2.5.3. PTIO Radical Scavenging Assay

The PTIO radical scavenging assay was assessed, referring to previously published methods, with some modifications [22]. In total, 100 μL of PFSO (0.625, 1.25, 2.5, 5, 10, and 20 mg/mL) at various concentrations dissolved in absolute ethanol was dispensed into a 96-well plate and mixed with 100 μL of PTIO–absolute ethanol (0.15 mg/mL). After the reaction solution was thoroughly mixed, it was incubated in the dark for 30 min. OD_557nm_ was evaluated, and Trolox was employed as a positive control.

#### 2.5.4. ABTS Radical Scavenging Assay

The ABTS radical scavenging assay was evaluated, as reported by Pei et al. [23]. The reaction was carried out in the dark at room temperature for 30 min with 50 μL of PFSO and 150 μL of diluted ABTS solution. OD_734nm_ was evaluated, and Trolox was employed as a positive control.

### 2.6. Assessment of Tyrosinase Inhibition Mechanism

#### 2.6.1. Mushroom Tyrosinase Activity Assay

The activity of mushroom tyrosinase was assessed using an assay, following a previously published method with slight modifications [24]. Activity assays were carried out in a 10 mM phosphate-buffered solution (PBS) of pH 6.8. For each experimental group, PFSOs (with final concentrations of 0.1, 0.25, 0.5, 0.75, 1, 1.25, and 1.5 mg/mL) dissolved in PBS were dispensed into a 96-well plate and mixed with 200 μL of the mushroom tyrosinase enzyme solution (200 units/mL). Subsequently, the mixture received 300 μL of PBS and 400 μL of 0.5 mM L-DOPA. Following a 30 min incubation period at 37 °C for 30 min to promote the enzyme reaction, OD_475nm_ was evaluated, and Trolox was employed as a positive control.

#### 2.6.2. Analysis of Inhibitory Kinetics

Lineweaver–Burk plots were used to examine the inhibitory kinetics of tyrosinase by PFSO [25]. Initial rates were determined at varying concentrations of L-DOPA in the presence of different concentrations of PFSO and were analyzed by a Lineweaver–Burk plot to determine the type of inhibition.

(1) $$\frac{1}{V}=\frac{K_{m}}{V_{max}}\times \frac{1}{\left[S\right]}+\frac{1}{V_{\max}}$$

Here, K_m_ is the Michaelis–Menten constant, V_max_ is the maximum reaction velocity of tyrosinase, and [S] is the concentration of the substrate.

#### 2.6.3. Copper Ions Chelation Assay

Referring to the method reported [26], UV spectrometry was used to evaluate the metal chelating properties of PFSO. In total, 2700 μL of copper ion solutions (50, 100, 150, and 200 μM) of various concentrations and PFSO (0.5 mg/mL) solutions were mixed to react at room temperature for 10 min. The absorbance was evaluated at a wavelength of 200–400 nm using an ultraviolet spectrophotometer (TU-1950, PERSEE, Beijing, China).

#### 2.6.4. Fluorescence Spectroscopy Assay

Building on the previous report [27], the effect of PFSO on the fluorescence intensity of tyrosinase was investigated. In total, 20 μL of different concentrations of PFSO solutions was added to 200 U/mL tyrosinase solution (180 μL) and kept for 10 min to equilibrate. The fluorescence spectroscopy was measured at 298, 304, and 310 K using a multifunctional microplate reader (Varioskan LUX, ThermoFisher, Waltham, MA, USA), respectively. The emission wavelength was measured between 290 and 450 nm, whereas the excitation wavelength was set at 280 nm. The Stern–Volmer equation was assessed to determine the fluorescence quenching type.

(2) $$\frac{F_{0}}{F}=1+K_{\mathrm{sv}}\left[Q\right]$$

where F and F_0_ represent the tyrosinase fluorescence intensity in the presence or absence of PFSO, [Q] represents PFSO concentration, and K_sv_ represents the quenching constant.

#### 2.6.5. Synchronous Fluorescence Measurement

The synchronized fluorescence assay was measured according to the previous methods [28]. Synchronized fluorescence of tyrosinase was studied upon the addition of PFSO at a temperature of 303 K. Δλ was fixed at 15 nm (for Tyr) and 60 nm (for Trp), and the scanning range was 290 to 400 nm.

#### 2.6.6. Molecular Docking

To investigate the interaction between PFSO and tyrosinase, a molecular model study was considered using AutodockTools (ADT) 1.5.6. The 3D structure of tyrosinase (PDB ID: 2Y9X) and its ligands (linoleic acid (CID: 5280450) and β-sitosterol (CID: 222284) of PFSO) were sourced from the PubChem Data Bank (https://pubchem.ncbi.nlm.nih.gov/, accessed on 17 January 2026) and the RCSB Protein Data Bank (http://www.rcsb.org/, accessed on 17 January 2026) [29], respectively. Before docking, tyrosinase was prepared by removing water molecules and adding hydrogenation using ADT. A grid box (126 Å × 126 Å × 126 Å, 0.85 Å spacing) was centered on the geometric center of tyrosinase (coordinates: X, Y, Z). The optimal binding models were generated using PyMOL 2.6 software.

#### 2.6.7. Cell Culture

B16F10 mouse melanoma cells (purchased from the Cell Bank of the Chinese Academy of Sciences, Shanghai, China) were cultured in RPMI 1640 medium supplemented with 10% fetal bovine serum (FBS, Gibco) and 1% penicillin–streptomycin solution. The cells were cultured in a humidified incubator at 37 °C with 5% CO_2_ and subcultured once every 2–3 days.

#### 2.6.8. Cell Viability

Briefly, B16F10 cells were seeded into a 96-well plate at a density of 5000 cells per well. Cells were treated with various concentrations of PFSO (25, 50, 100, 250, and 500 μg/mL) diluted in media and cultured for 48 h. The CCK-8 working solution was prepared according to the manufacturer’s instructions (Biyuntian Biotechnology Co., Ltd., Shanghai, China). Subsequently, each well was filled with 100 μL of the working solution and incubated for four hours at 37 °C. The absorbance was recorded at 450 nm.

#### 2.6.9. Intracellular Melanin Content Assay

Melanin content was measured and evaluated according to a previously published method [30]. Briefly, a 96-well plate was seeded with 5000 B16F10 cells per well. Cells treated with various concentrations of PFSO (25, 50, 100, 250, and 500 μg/mL) were incubated with 1 μM α-MSH for 48 h. Cells were washed twice with PBS, detached using trypsin, and centrifuged at 2000× *g* for 10 min. The resulting pellet was lysed in 250 μL of 1 M NaOH by heating at 80 °C for 30 min. OD_490nm_ was evaluated.

#### 2.6.10. Intracellular Tyrosinase Activity Assay

Intracellular tyrosinase activity was assessed according to established protocols [31]. A 96-well plate was seeded with 5000 B16F10 cells per well. Cells treated with various concentrations of PFSO (25, 50, 100, 250, and 500 μg/mL) were incubated with 1 μM α-MSH for 48 h. After washing twice with PBS, 100 μL of a 0.5% (*w*/*v*) sodium deoxycholate solution was added to each well, and cells were lysed on ice for 15 min to obtain a tyrosinase-containing extract. Following preincubation at 37 °C, 50 μL of a 0.3% (*w*/*v*) L-DOPA solution was added to proceed at 37 °C for 10 min. OD_475nm_ was evaluated.

### 2.7. Zebrafish Study

#### 2.7.1. Determination of Safe Concentration of PFSO in Zebrafish

To ascertain the maximum safe concentration of PFSO for zebrafish embryos, a 96-well plate was employed. At 24 h post-fertilization (hpf), one embryo was placed in each well and subjected to a Holt Buffer with various PFSO concentrations. The test included eight treatment groups and one control group, each with three replicates, and embryos were grown at 28.5 °C until observation at 72 hpf.

#### 2.7.2. Antioxidant Activity Assay in Zebrafish

To evaluate the antioxidant activity of PFSO on zebrafish, ROS in zebrafish were determined using the cell-permeable fluorescent probe H_2_DCF-DA (2.5 μM, 1 mL per well) [32]. The zebrafish embryos at 24 hpf were exposed to p-hydroxyacetophenone (p-HAP, 50 μM) to induce oxidative stress and then treated with the Holt Buffer containing PFSO (0.3125–5 mg/mL) in a 28.5 °C incubator. At 72 hpf, the embryos were washed three times with the Holt Buffer. The treated embryos were incubated with H_2_DCF-DA for 1 h in the dark at 28 °C. After three additional washes, zebrafish embryos were observed under a Zeiss Axio Imager.2 microscope imaging system (5× objective, FITC channel; Hamburg, Germany). The fluorescence intensity of single zebrafish embryos was quantified by Image J software (Version 13.0.6.) (National Institutes of Health, Bethesda, MD, USA). Embryos treated with p-HAP alone were used as the positive control. The experiment was repeated three times.

#### 2.7.3. Zebrafish Surface Melanin Scale Assay

The surface melanin scale of zebrafish was assessed by referring to previous methods [33]. The Holt buffer with different concentrations of PFSO was used as the blank control in a 24-well plate containing 15 embryos per well at 24 hpf. Ten zebrafish embryos per group were photographed at 72 hpf using the Zeiss Axio Imager.2 microscope imaging equipment to record melanin distribution from a top-down view. Melanin from the head to one-quarter of the yolk sac was quantified using ImageJ software. A region of interest was defined to obtain the integrated density, and the average pixel values were compared across different treatment groups to assess the melanin area size.

#### 2.7.4. Zebrafish Melanin Content Assay

The melanin content of zebrafish was assessed in accordance with a previous report [34]. In total, 15 embryos per well at 24 hpf were placed in a 24-well plate and treated with a Holt Buffer containing various concentrations of PFSO. The Holt Buffer was used as a blank control. Zebrafish embryos at 72 hpf were washed three times with the Holt Buffer and moved to 1.5 mL centrifuge tubes. Each tube received 250 μL of the previously stated total protein extraction solution, and the embryos were homogenized with a grinding rod until no discernible clumps remained. The samples were then centrifuged at 10,000 rpm for 5 min at 4 °C. Following centrifugation, the supernatant was separated for subsequent use, and the black precipitate was preserved. The black precipitate was dissolved in 60 μL of 1 M NaOH solution and incubated at 85 °C until the melanin was completely dissolved. The OD_450nm_ was recorded.

#### 2.7.5. Zebrafish Tyrosinase Activity Assay

The supernatant from the melanin content test was used to assess tyrosinase activity (Section 2.7.4). Specifically, 100 µL of the supernatant was added to 100 µL of 0.5 mM L-DOPA solution in a 96-well plate. The reaction mixture was incubated at 37 °C for 30 min, and the absorbance was recorded at 475 nm.

#### 2.7.6. RNA Isolation and Complementary DNA Synthesis

Total RNA was isolated from 72 hpf embryos in each group using a Total RNA Column Extraction Kit, following the manufacturer’s instructions. Reverse transcription was carried out using the HiScript III RT SuperMix for qPCR (+gDNA wiper) (Vazyme, #R323). This kit contained all necessary components for genomic DNA removal and cDNA synthesis, enabling the reaction to be performed by simply adding the template RNA.

#### 2.7.7. Quantitative Real-Time Polymerase Chain Reaction (RT-qPCR)

RT-qPCR was carried out using ChamQ Universal SYBR qPCR Master Mix (Vazyme, Nanjing, China) with self-designed primers. Amplification was performed on a CFX96 Touch Real-Time PCR Detection System (Bio-Rad, Hercules, CA, USA), with each RNA sample being analyzed in triplicate. β-actin was used as the internal reference gene to quantify relative gene expression using the 2^–ΔΔCt^ technique. All primers were independently designed and validated prior to use.

### 2.8. Statistical Analysis

All experiments were performed in triplicate and reported as the mean ± standard deviation. Statistical analyses of cell experiments and zebrafish experiments were performed using GraphPad Prism 8.0 (GraphPad Software, San Diego, CA, USA). Statistical analyses of the remaining part were conducted using Origin 2021 (OriginLab Corporation, Northampton, MA, USA) and SPSS 27 (IBM Corporation, Armonk, NY, USA).

## 3. Results and Discussion

### 3.1. Extraction Yield of PFSO

The method of oil extraction is one of the most critical steps to assess the quality and quantity of oil extracted [35]. Traditional methods present certain drawbacks: traditional pressing may lead to oil quality deterioration due to high temperatures [10], while Soxhlet extraction poses potential health risks from residual organic solvents [11]. In contrast, SC-CO_2_ extraction effectively overcomes these limitations. A single-factor experiment was conducted based on the conditions listed in Table 1. The results of the single-factor experiments are presented in Figure S1. The extraction yields initially increased and then decreased with rising temperature and pressure. In contrast, extending the dynamic and static extraction times led to an initial increase in yield, which eventually plateaued. Based on the orthogonal array design (Table S1), it was concluded that the optimum conditions for obtaining the highest oil extraction yield were the extraction temperature of 60 °C, the extraction pressure of 30 MPa, the dynamic extraction time of 105 min and the static extraction time of 100 min (A_3_B_2_C_3_D_2_). To validate the model, three verification experiments were conducted under these optimal conditions. The average oil extraction yield was 24.96% with a standard deviation of 0.002, confirming that the model is reliable for predicting the optimal extraction conditions.

### 3.2. Fatty Acid Composition of PFSO

The fatty acid composition is an important indicator for evaluating the bioactivity of oils. Unsaturated fatty acids provide various health benefits, including antioxidant effects, modulation of inflammatory responses, treatment of cardiovascular and neurological disorders [36], lipid-lowering activity [37], etc. In this study, the fatty acid composition of PFSO obtained by SC-CO_2_ extraction is presented in Table 2 and Figure S2. A total of 19 fatty acids were identified in PFSO. This indicated that the SC-CO_2_ extraction method retained more bioactive components in the oil. The PFSO samples contained high levels of unsaturated fatty acids (88.03%), which were substantially higher than other common edible oils sold on the market, such as rapeseed oil (≈52–53%) [38]. Notably, the SC-CO_2_-extracted PFSO was particularly rich in linoleic acid (74.40%) and contained 12.42% oleic acid. Linoleic acid is known to inhibit tyrosinase activity and reduce melanin production, suggesting a potential skin-whitening effect [39]. Additionally, a small amount of myristic acid (0.05%), a compound valued in cosmetics for its skin-moisturizing properties, further indicates the potential of PFSO for the cosmetic industry [40]. Notably, α-linolenic acid, an essential ω-3 polyunsaturated fatty acid for humans, which exhibited anti-inflammatory properties and reduced triglyceride levels [41], was detected at 0.51% in SC-CO_2_-extracted PFSO.

### 3.3. Content of Active Ingredients of PFSO

In addition to fatty acid composition, the contents of polyphenols and tocopherols also exerted a significant influence on the bioactivity of PFSO. Therefore, the TFC, β-carotene, TPC and Ve contents in PFSO were determined. The TFC, with anti-inflammatory and antioxidant activities, contributed to the overall antioxidant capacity of PFSO [42], enhancing radical scavenging. The standard curve was constructed using different concentrations of rutin standard at y = 8.39381x − 0.01577 with R^2^ = 0.998. The TFC of PFSO reached 1.89 mg/g. In addition, flavonoids demonstrated effective inhibition of tyrosinase activity [43].

As a well-recognized lipophilic antioxidant, β-carotene exerted a pronounced inhibitory effect on lipid oxidation [44]. To quantify its content, the calibration curve for the β-carotene standard was y = 0.23982x + 0.01402, R^2^ = 0.997. Based on this calibration, the β-carotene content in PFSO was determined to be 4.45 μg/g.

To quantify the TPC in PFSO, the calibration curve of the gallic acid standard was established as y = 0.00204x − 0.0179 with R^2^ = 0.999. Based on this calibration, the TPC in PFSO was determined to be 84.37 mg GAE/kg, which was higher than the PFSO extracted by Lee et al. [45]. Phenolic compounds exhibited diverse physiological activities, including antioxidant, anti-inflammatory, and antimicrobial functions [46]. The exploration of polyphenol-rich vegetable oils and their associated health-promoting properties has become a prominent research focus. Additionally, polyphenols have also been demonstrated to significantly inhibit the activity of tyrosinase [47], which provides potential for subsequent research on inhibiting tyrosinase.

Tocopherols exist as four isoforms: α-, β-, γ-, and δ-tocopherol. Although these tocopherols exhibit comparable antioxidant activities in vitro, α-tocopherol demonstrates the highest vitamin E activity in vivo [48]. γ-Tocopherol, frequently present in food sources, possesses distinctive capabilities in scavenging nitrogen-centered free radicals and exerting anti-inflammatory effects [49]. δ-Tocopherol has recently emerged as a research focus, displaying promising potential in anticancer applications [50]. In this study, the concentrations of four tocopherol isomers in PFSO were quantified. The calibration curve for γ-tocopherol was y = 48.52544x + 0.00042, with R^2^ = 0.999, and the calibration curve for δ-tocopherol was expressed as y = 58.10039x + 0.0005, with R^2^ = 0.999 (Figure S3). Neither α-tocopherol nor β-tocopherol was detected in PFSO. Only γ-tocopherol and δ-tocopherol isomers were detected, with concentrations of 5.82 mg/100 g and 4.51 mg/100 g, respectively. The total vitamin E activity, expressed as α-tocopherol equivalents (α-TE), was 0.63 mg α-TE/100 g. Ve, known as tocopherol, exhibited a strong free radical scavenging capacity, antioxidant activity and retarded lipid oxidation [51]. In addition, Ve significantly enhanced the inhibition of tyrosinase, which was demonstrated in previous studies [52].

### 3.4. In Vitro Antioxidant Activity of PFSO

**Figure 1:**
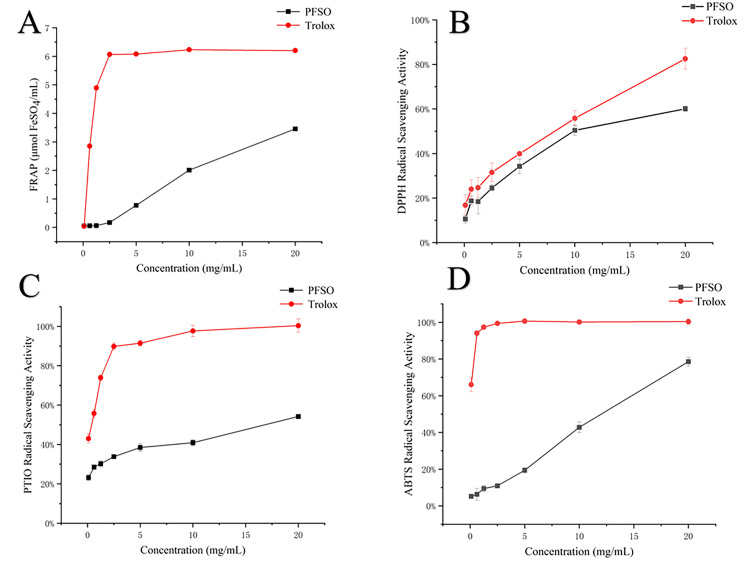
The results of the radical scavenging rate of PFSO: (**A**) the FRAP of PFSO; (**B**) DPPH radical scavenging rate of PFSO; (**C**) PTIO radical scavenging rate of PFSO; (**D**) ABTS radical scavenging rate of PFSO.

The in vitro antioxidant capacity of PFSO was evaluated using multiple assays, including DPPH, ABTS, and PTIO radical scavenging assays, as well as the FRAP (Ferric-Reducing Antioxidant Power) assay, with the results exhibited in Figure 1. All assays demonstrated a dose-dependent response within the tested concentration range (0.1–20 mg/mL). Notably, the FRAP value reached 3.46 μmol FeSO_4_/mL at the highest concentration (20 mg/mL), indicating a strong reduction capacity. The IC_50_ values for PFSO were determined to be 11.33 ± 0.87 mg/mL for ABTS, 12.74 ± 1.73 mg/mL for DPPH, and 27.00 ± 0.57 mg/mL for PTIO scavenging activities. When compared with data from the literature for other plant oils, the DPPH scavenging activity of PFSO (IC_50_ = 12.74 mg/mL) was superior to that of canola, sunflower, and groundnut oils, which exhibited less than 50% scavenging at unspecified concentrations [53]. Similarly, the FRAP value of PFSO (3.46 μmol FeSO_4_/mL) was higher than that of flaxseed oil (<2 μmol FeSO_4_/mL) [54], and its ABTS scavenging activity (IC_50_ = 11.33 mg/mL) exceeded that of Sesamum indicum L. oil (<20% scavenging). However, the PTIO scavenging capacity of PFSO (IC_50_ = 27.00 mg/mL) was lower than that of blackberry seed oil (IC_50_ < 10 mg/mL) [55]. In summary, PFSO exhibited lower free radical scavenging activity compared with the positive control (Trolox) and exhibited varying scavenging efficiencies against different free radicals. Given that a lower IC_50_ value indicates stronger antioxidant capacity, the overall radical scavenging activity of PFSO followed the order ABTS > DPPH > PTIO.

### 3.5. Tyrosinase Inhibition Mechanism of PFSO

#### 3.5.1. Mushroom Tyrosinase Activity of PFSO

**Figure 2:**
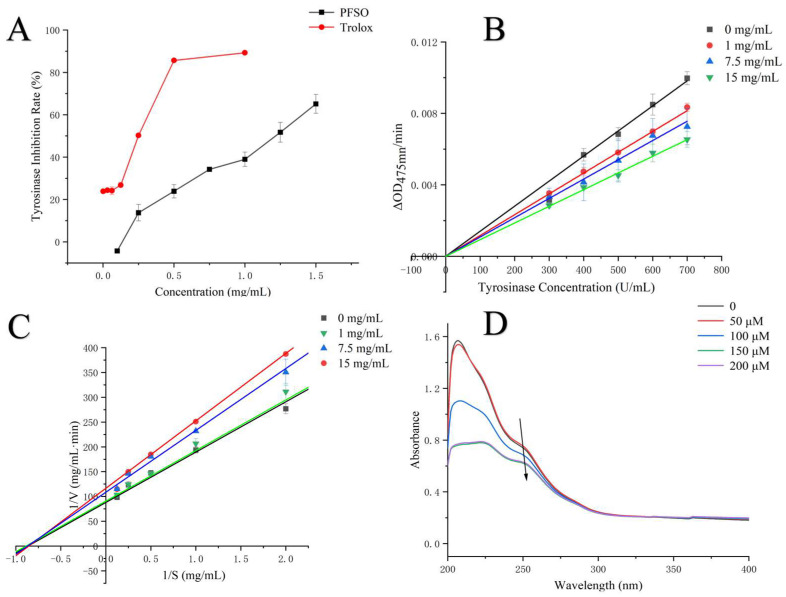
Inhibitory kinetics of tyrosinase by PFSO: (**A**) inhibitory effects of PFSO and trolox; (**B**) plots of *ν* (∆OD/min) vs. tyrosinase concentration; (**C**) Lineweaver–Burk plots; (**D**) copper-chelating ability of PFSO.

Melanin synthesis was predominantly regulated by tyrosinase. In this study, the effects of PFSO at varying concentrations on the tyrosinase-mediated oxidation of L-DOPA were investigated. Tyrosinase activity was inhibited by PFSO in a dose-dependent manner, as shown in Figure 2A. The IC_50_ value of PFSO on tyrosinase activity was calculated to be 1.12 ± 0.01 mg/mL, suggesting that PFSO was an effective inhibitor of tyrosinase.

#### 3.5.2. Kinetic Analysis of Tyrosinase

To investigate the reversibility of inhibition, reaction rates were plotted against enzyme concentrations at different PFSO levels. As shown in Figure 2B, all regression lines passed through the origin, suggesting that PFSO exhibited reversible inhibition toward tyrosinase, which is consistent with previously reported findings from Chai et al. on longan shell tannins [56].

Lineweaver–Burk double reciprocal plots (1/V versus 1/S) were constructed to determine the type of inhibition. As shown in Figure 2C, all regression lines intersected at one point in the second quadrant, indicating that PFSO exerted a mixed-type inhibition on tyrosinase activity, with the ability to bind to both the free enzyme and the enzyme–substrate complex [25]. The corresponding kinetic parameters, K_m_ and V_max_, were calculated according to the equation, as listed in Table 3. K_m_ increased, and V_max_ decreasedwith increasing concentrations of PFSO.

#### 3.5.3. Copper Ion Chelation of Tyrosinase

Tyrosinase contains two copper ions at its active site, which play a crucial role in catalyzing the enzymatic reaction [57]. Therefore, the chelation ability of copper ions significantly influences the activity of tyrosinase. As shown in Figure 2D, the UV absorption spectrum of the Cu^2+^ solution showed a peak at 247 nm. Upon addition of PFSO, this peak decreased in intensity and exhibited a slight red shift (247 → 250 nm), indicating the formation of a complex between PFSO and copper ions [58].

#### 3.5.4. Fluorescence Spectroscopy of Tyrosinase

**Figure 3:**
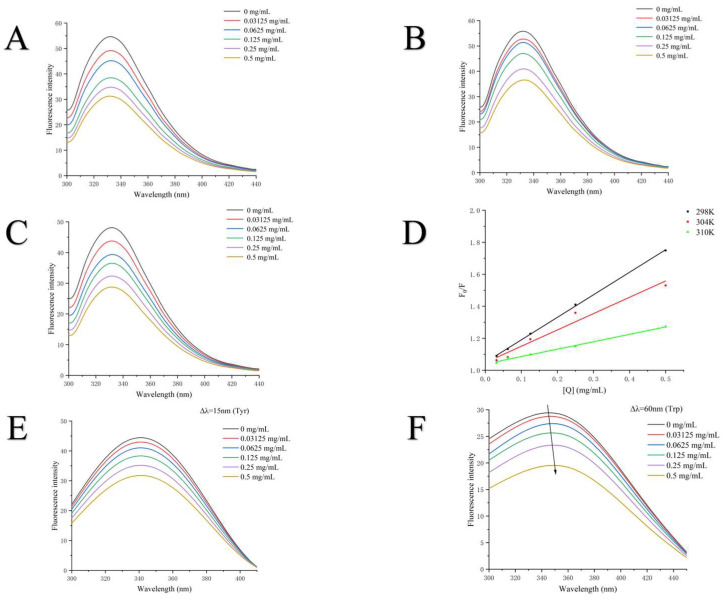
Fluorescence spectra of tyrosinase in the absence and presence of PFSO at different temperatures with various concentrations: (**A**) 298 K; (**B**) 304 K; and (**C**) 310 K. (**D**) Stern–Volmer plots for the fluorescence quenching of tyrosinase by PFSO at three different temperatures (298, 304, and 310 K). (**E**) Synchronous fluorescence spectra of tyrosinase in the absence and presence of PFSO (Δ*λ* = 15 nm). (**F**) Tyrosinase synchronous fluorescence spectra with and without PFSO (Δ*λ* = 60 nm). Tyrosinase fluorescence intensities in the presence and absence of PFSO are shown by the symbols F_0_ and F, respectively.

The intrinsic fluorescence of tyrosinase, primarily contributed to by tryptophan (Trp) residues, was employed in this study to assess its interaction with PFSO [59]. In this experiment, the fluorescence emission spectra of tyrosinase were measured at various concentrations of PFSO under an excitation wavelength of 280 nm to investigate its conformational changes. As shown in Figure 3A–C, a maximum absorption peak was observed at a wavelength of 332 nm. As the concentration of PFSO increased, the fluorescence intensity decreased significantly, indicating that PFSO interacted with tyrosinase and quenched its fluorescence intensity, which was consistent with prior reports [60]. Additionally, the absence of a significant shift in wavelength suggested that PFSO did not induce a conformational change in the enzyme, but potentially altered the microenvironment around the fluorophore through interaction [61]. To further elucidate the fluorescence quenching mechanism of PFSO toward tyrosinase, the Stern–Volmer equation was applied to analyze the fluorescence data. As shown in Figure 3D, the Stern–Volmer plot exhibited good linearity at different temperatures, suggesting that PFSO quenched tyrosinase fluorescence through a single quenching mechanism. The values of *K*_sv_ and the line slopes decreased with rising temperatures (298, 304, and 310 K), which suggested that the quenching was static, likely due to the formation of a PFSO–tyrosinase complex, the stability of which decreased at higher temperatures [61].

The synchronous fluorescence spectra were measured to monitor the microenvironmental changes in tyrosine (Tyr) and tryptophan (Trp) residues in tyrosinase. The maximum fluorescence intensity of Tyr residues decreased progressively with increasing concentrations of PFSO, as shown in Figure 3E, without a shift in the maximum emission wavelength, indicating that the microenvironment surrounding Tyr residues was not affected by PFSO. In contrast, the maximum emission wavelength of Trp residues exhibited a slight red shift (Figure 3F), suggesting that PFSO could change the microenvironment around Trp residues and increase its hydrophobicity, similar to previous studies [25].

#### 3.5.5. Molecular Modeling Study

**Figure 4:**
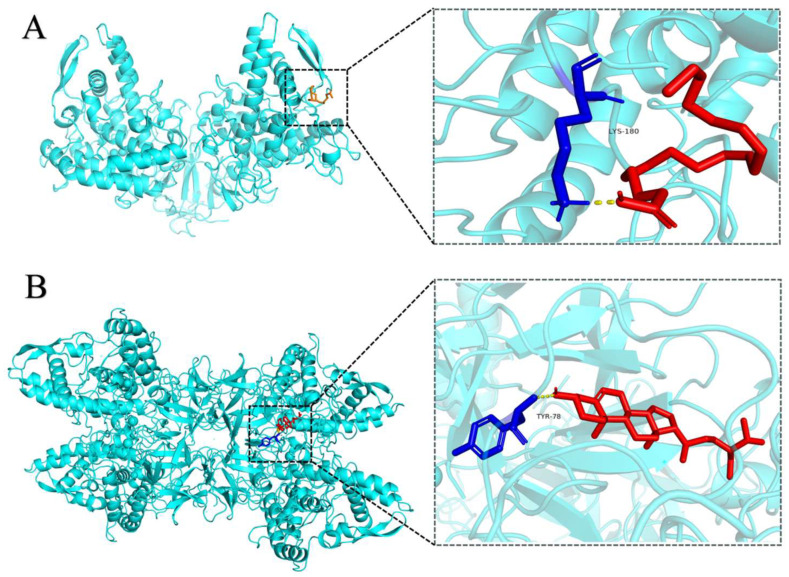
Molecular docking of ligands with tyrosinase. (**A**) 3D perspective views of the optimal binding region for the docking of linoleic acid to the tyrosinase molecule. (**B**) 3D perspective views of the optimal binding region for the docking of β-sitosterol to the tyrosinase molecule. The yellow dashed line represents the hydrogen bonds.

**Figure 5:**
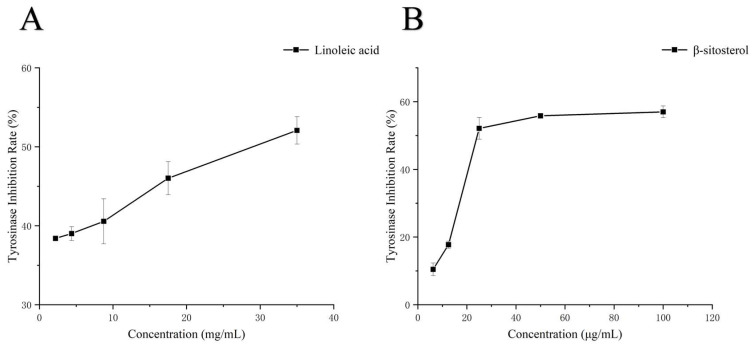
Tyrosinase-inhibitory activity of (**A**) linoleic acid and (**B**) β-sitosterol.

Based on the results of compound analysis (Table S2), linoleic acid and β-sitosterol were identified as having potential tyrosinase-inhibitory activity in PFSO. Therefore, linoleic acid and β-sitosterol served as representative ligands for molecular docking simulations. The docking results are shown in Figure 4. The docking energy between tyrosinase and linoleic acid was −3.21 kcal/mol, forming a hydrogen bond with the LYS180 residue and the docking energy between tyrosinase and β-sitosterol was −8.16 kcal/mol, forming a hydrogen bond with the TYR78 residue. In addition, hydrophobic interactions are shown in Table S3. Thus, hydrogen bond and hydrophobic interactions served as the primary driving forces for the binding of linoleic acid and β-sitosterol to tyrosinase. Although the binding site was not the critical Cu^2+^ site in tyrosinase, changes in the microenvironment surrounding certain amino acid residues were induced, affecting the enzymatic activity of tyrosinase, which was in agreement with earlier observations by Yu et al., who demonstrated that asparagus polyphenols inhibit tyrosinase activity through a comparable pathway [61]. Although molecular docking analyses suggested the potential bioactivity of linoleic acid and β-sitosterol, experimental validation was required. Accordingly, authentic standards of both compounds were purchased to evaluate their inhibitory effects on mushroom tyrosinase activity. As shown in Figure 5, linoleic acid and β-sitosterol exhibited notable tyrosinase-inhibitory activity (IC_50_ = 29.44 mg/mL and 46.43 μg/mL, respectively), which is in line with the data from molecular docking analyses.

#### 3.5.6. Cytotoxicity of the PFSO and Effects of PFSO on Melanin Production in α-MSH-Stimulated B16F10 Cells

**Figure 6:**
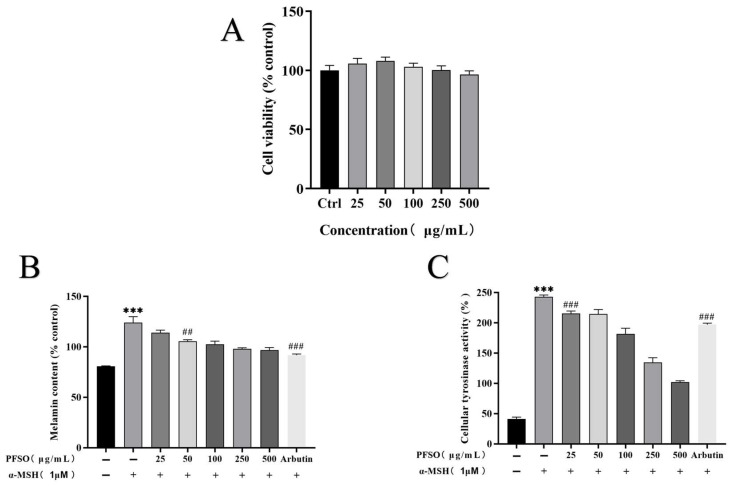
Effect of PFSO on cell viability and inhibition of melanin production in α-MSH-treated B16F10 cells. (**A**) Cell viability was measured after B16F10 cells were treated with various concentrations of PFSO (25–500 μg/mL) for 48 h. (**B**) Melanin content. (**C**) Intracellular tyrosinase activity. *** *p* < 0.001 compared to the untreated control group; ## *p* < 0.01; and ### *p* < 0.001 compared to the α-MSH-treated group.

In light of the favorable results obtained from cell-free assays demonstrating the tyrosinase inhibitory and antioxidant potential of PFSO, a critical cytotoxicity evaluation was subsequently performed. Given the safety concerns associated with various antimelanogenic agents, this assessment was essential to comprehensively characterize the safety profile of PFSO. No cytotoxic effects on B16F10 cells were observed across the entire range of PFSO concentrations tested (Figure 6A). Accordingly, only non-cytotoxic concentrations of PFSO were employed in subsequent experiments. To further validate the whitening potential of PFSO at the cellular level, its antimelanogenic effects were evaluated in α-MSH-stimulated B16F10 murine melanoma cells. As expected, α-MSH treatment significantly increased melanin production compared with the untreated control group (*p* < 0.001), which confirmed the successful generation of the pigmentation model. Notably, PFSO (25–500 μg/mL) dose-dependently reduced melanin content compared with the α-MSH-treated group (*p* < 0.01), as shown in Figure 6B. The effects of PFSO on intracellular tyrosinase activity in B16F10 cells were further evaluated. α-MSH stimulation significantly increased tyrosinase activity (*p* < 0.001), consistent with enhanced melanogenesis. Treatment with PFSO (25 μg/mL) significantly attenuated the α-MSH-induced upregulation of intracellular tyrosinase activity (*p* < 0.001). In addition, PFSO dose-dependently reduced intracellular tyrosinase activity compared with the α-MSH-treated group (Figure 6C). Arbutin, employed as a positive control [62], markedly suppressed melanin synthesis and intracellular tyrosinase activity (*p* < 0.001), consistent with its established skin-whitening efficacy. These results suggest that PFSO effectively inhibits tyrosinase activity and melanin production at the cellular level.

### 3.6. Effects of PFSO on Zebrafish

#### 3.6.1. In Vivo Antioxidant Effect of PFSO

**Figure 7:**
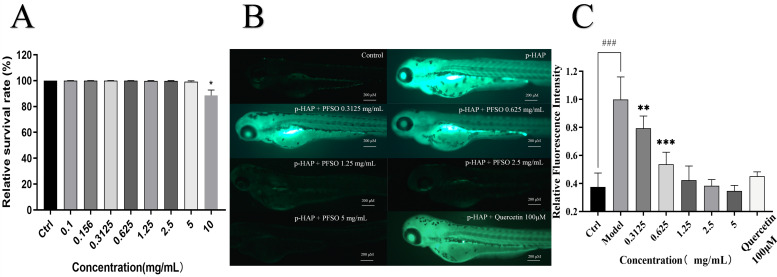
The maximum safe concentration of PFSO in zebrafish and the level of ROS in zebrafish in a p-HAP oxidative stress model with PFSO treatment: (**A**) the maximum safe concentration of PFSO; (**B**) representative fluorescence images of ROS detection; (**C**) fluorescence intensity quantification results. ### *p* < 0.001, compared with the control group. * *p* < 0.05, ** *p* < 0.01, and *** *p* < 0.001, compared with the model group.

The maximal safe dose of PFSO in the zebrafish model was found to be 5 mg/mL (Figure 7A). ROS can activate melanogenesis-associated transcription factors, which upregulate tyrosinase expression [63]. Accordingly, reducing the production of ROS was also an approach to skin-whitening. Compared with the blank control group, the fluorescence intensity of the model group treated with p-HAP was significantly enhanced, confirming that the oxidative stress model was successfully established (Figure 7B,C). In comparison with the model group, the fluorescence intensity in the PFSO-treated groups was effectively reduced in a dose-dependent manner. When the concentration of PFSO was only 0.625 mg/mL, the fluorescence intensity and ROS production were reduced, indicating that PFSO effectively reduces p-HAP-induced ROS production. Notably, a significant reduction in ROS production and fluorescence intensity was observed even at the lowest PFSO concentration tested (0.625 mg/mL), demonstrating the potential in vivo antioxidant capacity of PFSO.

#### 3.6.2. Melanin Content and Tyrosinase Activity in Zebrafish

**Figure 8:**
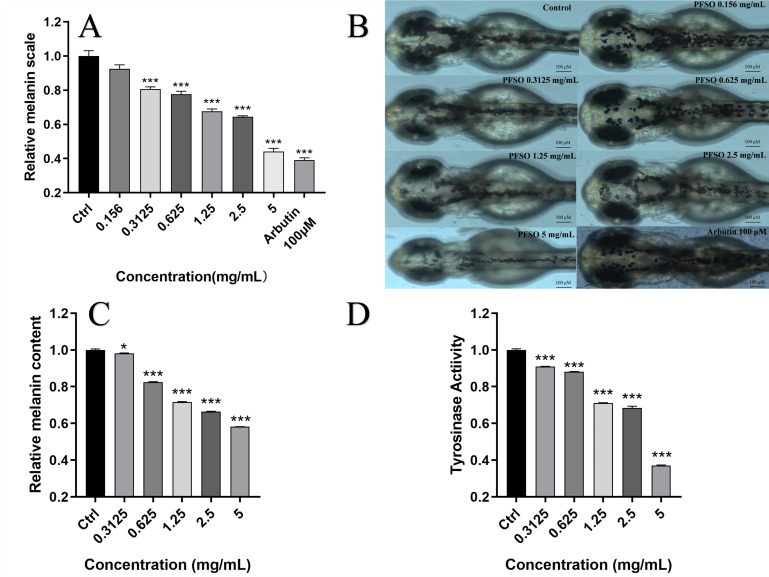
The impact of PFSO at various concentrations on the accumulation of zebrafish melanin: (**A**) surface melanin scale of zebrafish embryos; (**B**) surface melanin scale of zebrafish images with or without PFSO treatment; (**C**) relative melanin content of zebrafish embryos; (**D**) relative activity of tyrosinase in zebrafish embryos. * *p* < 0.05, and *** *p* < 0.001, compared with the control group.

Compared with the control group, PFSO treatment significantly decreased the surface melanin scale of zebrafish in a dose-dependent manner, as shown in Figure 8A,B. Notably, the significant inhibitory effect on melanin deposition was observed at a concentration as low as 0.3125 mg/mL (*p* < 0.001).

As shown in Figure 8C, the inhibitory effect of PFSO on melanogenesis was confirmed quantitatively, with melanin content decreasing dose-dependently. While low concentrations had a modest effect, significant suppression of melanin content (*p* < 0.001) was achieved at concentrations above 0.625 mg/mL. Following PFSO treatment, the reduction in melanin accumulation was attributed to a decrease in melanin content, as observed for the surface melanin scale of zebrafish. At a maximum safe concentration of 5 mg/mL, the 42% inhibition rate on melanin content was caused by PFSO. Owing to its edibility and natural origin, PFSO demonstrates great potential as a safe and distinctive alternative to conventional pigmentation regulators available on the market.

As shown in Figure 8D, consistent with the observed reduction in pigmentation, the tyrosinase activity in zebrafish embryos was inhibited by PFSO in a dose-dependent manner. Compared with the control group, a significant reduction in tyrosinase activity was detected at concentrations of only 0.3125 mg/mL, aligning with the inhibitory pattern observed for mushroom tyrosinase in vitro.

#### 3.6.3. Downregulation of Whitening-Related Gene Expression in Zebrafish

**Figure 9:**
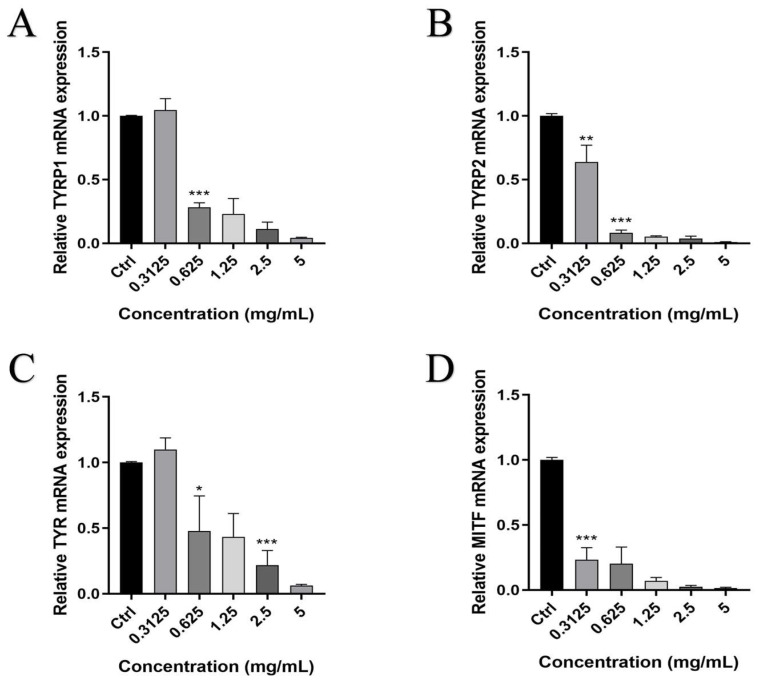
Relative gene expression levels of whitening-related genes: (**A**) TYRP1; (**B**) TYRP2; (**C**) TYR; and (**D**) MITF. * *p* < 0.05 ** *p* < 0.01 and *** *p* < 0.001, compared with the control group.

Melanogenesis in zebrafish is regulated by the genes TYRP1, TYRP2, TYR, and MITF [64]. The self-designed primers sequences were listed in Table 4. qRT-PCR was used to assess the expression levels of TYRP1, TYRP2, TYR, and MITF genes to identify the relationship between the decrease in tyrosinase activity, reduced melanin content, and the expression of melanogenesis-related genes. As shown in Figure 9, the expression of TYRP1, TYRP2, TYR, and MITF was inhibited by treatment with PFSO, which was closely associated with the downregulation of gene expression. The expression levels of TYRP1, TYRP2, TYR, and MITF were reduced by PFSO treatment in a dose-dependent manner. A significant inhibitory impact on the expression levels of TYRP2 and MITF was observed at a PFSO concentration of only 0.3125 mg/mL, whereas significant downregulation of TYRP1 and TYR required a higher concentration (0.625 mg/mL). These results indicate that PFSO could suppress melanogenesis by downregulating TYRP1, TYRP2, TYR, and MITF melanogenesis-related gene expressions.

**Table 4:** Self-designed primer sequences.

Gene Name	Front Primer Sequence (5′-3′)	Rear Primer Sequence (3′-5′)
β-actin	GCCGTGACCTGACTGACTAC	GGGCACCTGAACCTCTCATT
TYRP1 TYRP2 TYR MITF	TGCAACAGTACGGAGAGCAG AACGCACTGGATCTCAGCAA AATCGCATCGGTTTTCGCAG GTTCGAGAGCTACCAGAGGC	GTTCAGGAACAGGTGAGCCA GCGTGTCTCTGACGGAGTAG CGGATGAGATCAGCGTGTCA GTAATCCACCGACGCCTTCA

## 4. Conclusions

This study systematically explored the interaction of *Passiflora edulis f. edulis* seed oil and tyrosinase. PFSO was extracted using supercritical CO_2_ technology optimized by orthogonal design. PFSO exhibited a yield of 24.96%, with a high content of unsaturated fatty acids (88.03%, mainly linoleic acid at 74.40%) and considerable levels of bioactive compounds, including phenolics, flavonoids, β-carotene, and tocopherols, which collectively contributed to its strong antioxidant capacity and great radical scavenging. The kinetic analysis showed that PFSO had significant inhibitory activity on tyrosinase in a reversible and mixed-type manner with an IC_50_ value of 1.12 ± 0.01 mg/mL. Molecular docking confirmed that linoleic acid bound to tyrosinase at LYS180 and the β-sitosterol of PFSO bound to tyrosinase at TYR78, further supporting its inhibitory effect. Further validation experiments demonstrated that linoleic acid, the predominant constituent, exhibited only weak tyrosinase inhibitory activity (IC_50_ = 29.44 mg/mL), whereas β-sitosterol, identified as a key bioactive component, exerted pronounced inhibition of tyrosinase activity (IC_50_ = 46.43 μg/mL). In vitro assays demonstrated the PFSO’s efficacy in significantly reducing the melanin content and tyrosinase activity in α-MSH-stimulated B16F10 cells. In vivo experiments using zebrafish further demonstrated that PFSO exhibited potent antioxidant and depigmenting activities. PFSO effectively suppressed ROS production induced by p-HAP, reduced surface melanin deposition, and inhibited tyrosinase activity in a dose-dependent manner. Importantly, PFSO downregulated the expression of melanogenesis-related genes (TYRP1, TYRP2, TYR, and MITF), confirming its ability to suppress melanogenesis at the molecular level. Collectively, these findings provide comprehensive evidence of PFSO as a natural, safe, and effective antioxidant and tyrosinase inhibitor, integrating extraction optimization, multicomponent characterization, multimodal inhibition analysis, and in vivo validation.

## Figures and Tables

**Table 1:** Summary of SC-CO_2_ extraction for single-factor experiment.

Factor	(A) Extraction Temperature (°C)	(B) Extraction Pressure (MPa)	(C) Dynamic Extraction Time (min)	(D) Static Extraction Time (min)
1	40	15	0	40
2	45	20	35	60
3	50	25	70	80
4	55	30	105	100
5	60	35	140	120

**Table 2:** Fatty acid composition of PFSO.

Types of Fatty Acids	SC-CO_2_ Extraction (%)
Caprylic acid (C8:0)	0.03 ± 0.02
Myristic acid (C14:0)	0.05 ± 0.01
Pentadecanoic acid (C15:0)	0.02 ± 0.00
Palmitic acid (C16:0)	9.62 ± 0.15
Palmitoleic acid (C16:1)	0.23 ± 0.02
Heptadecanoic acid (C17:0)	0.09 ± 0.01
Heptadecenoic acid (C17:1)	0.04 ± 0.00
Stearic acid (C18:0)	1.89 ± 0.08
Oleic acid (C18:1)	12.42 ± 0.63
Linoleic acid (C18:2)	74.40 ± 0.87
γ-linolenic acid (C18:3 (n-6))	0.25 ± 0.09
α-linolenic acid (C18:3 (n-3))	0.51 ± 0.04
Arachidic acid (C20:0)	0.12 ± 0.01
Eicosenoic acid (C20:1)	0.11 ± 0.01
Eicosadienoic acid (C20:2)	0.03 ± 0.00
Behenic acid (C22:0)	0.06 ± 0.01
Tricosanoic acid (C23:0)	0.03 ± 0.00
Lignoceric acid (C24:0)	0.05 ± 0.00
Docosahexaenoic acid (C22:6)	0.04 ± 0.01
Unsaturated fatty acids	88.03 ± 0.24
Monounsaturated fatty acids (MUFAs)	12.80 ± 0.64
Polyunsaturated fatty acids (PUFAs)	75.23 ± 0.88
Saturated fatty acids	11.97 ± 0.26
PUFA/MUFA	13.60 ± 0.33

**Table 3:** Tyrosinase kinetic characteristics at various PFSO concentrations.

PFSO (mg/mL)	K_m_ (mg/mL)	V_max_ (mg/L•min)
0	1.15	1.10 × 10^−2^
1 7.5 15	0.89 1.15 1.17	9.8 × 10^−3^ 9.2 × 10^−3^ 8.6 × 10^−3^

## Data Availability

The original contributions presented in this study are included in the article/Supplementary Material. Further inquiries can be directed to the corresponding author.

## Supplementary Materials

The following supporting information can be downloaded at https://www.mdpi.com/article/10.3390/foods15071246/s1. Table S1: SC-CO_2_ orthogonal array design. Table S2: GC-MS analysis of PFSO. Table S3: Molecular docking results of tyrosinase with linoleic acid and β-sitosterol. Figure S1: Single-factor analysis of SC-CO_2_ extraction of PFSO: (A) the effect of extraction temperature on the extraction yield; (B) the effect of extraction pressure on the extraction yield; (C) the effect of static extraction time on the extraction yield; (D) the effect of dynamic extraction time on the extraction yield. Figure S2: GC chromatograms of PFSO. Figure S3: Standard curves and liquid chromatograms of Ve in PFSO: (A) liquid chromatograms of different concentrations of Ve standards; (B) standard curve for γ-tocopherol; (C) standard curve for δ-tocopherol.

## Abbreviations

ABTS	2,2′-azino-bis(3-ethylbenzothiazoline)-6-sulphonic acid
α-MSH	α-melanocyte-stimulating hormone
α-TE	α-tocopherol equivalent
B16F10	B16F10 mouse melanoma cells
CCK-8	Cell counting kit-8
DPPH	2,2-Diphenyl-1-picrylhydrazyl
FBS	Fetal bovine serum
FRAP	Ferric-reducing antioxidant power
GC	Gas chromatography
H_2_DCFH-DA	2′,7′-dichlorodihydrofluoresceine diacetate
HPLC	High-performance liquid chromatography
L-DOPA	L-3,4-dihydroxyphenylalanine
MITF	Melanocyte-inducing transcription factor
OECD	Organization for Economic Cooperation and Development
PBS	Phosphate-buffered saline
PFSO	Passion fruit seed oil
p-HAP	p-hydroxyacetophenone
PTIO	2-Phenyl-4,4,5,5-tetramethylimidazoline-1-oxyl 3-Oxide
ROS	Reactive oxygen species
SC-CO_2_	Supercritical CO_2_ extraction
TFC	Total flavonoid content
TPC	Total phenolic content
Tyr	Tyrosine
TYR	Tyrosinase
TYRP1	Tyrosinase-related protein 1
TYRP2	Tyrosinase-related protein 2
Ve	Vitamin E
