# Supplementary material for: Passion Fruit Seed Oil as a Natural Tyrosinase Inhibitor: Extraction Optimization, Multi-Mechanism Elucidation, and Efficacy Validation in Zebrafish
Source: Foods. 2026 Apr 6;15(7):1246. doi: 10.3390/foods15071246 (PMC13073556; doi:10.3390/foods15071246)
Supplement: Supplementary file 1 [file foods-15-01246-s001.zip › foods-4234077-supplementary.pdf]

Supplementary data

Single factor experiments

The effect of extraction temperature on the extraction yield was analyzed at 40, 45, 50, 55 and 60 °C with the pressure of 30 MPa, the static extraction time of 80 min, and the dynamic extraction time of 70 min respectively. The effect of extraction pressure on the extraction yield was studied at 15, 20, 25, 30, 35 MPa with the extraction temperature of 50 °C, the static extraction time of 80 min, and the dynamic extraction time of 70 min. The effect of static extraction time on the extraction yield was analyzed at 40, 60, 80, 100, 120 min with the extraction temperature of 50 °C, the pressure of 30 MPa and the dynamic extraction time of 70 min. The effect of dynamic extraction time on the extraction yield was studied at 0, 35, 70, 105, 140 min with the extraction temperature of 50 °C, the pressure of 30 MPa and the static extraction time of 100 min respectively.

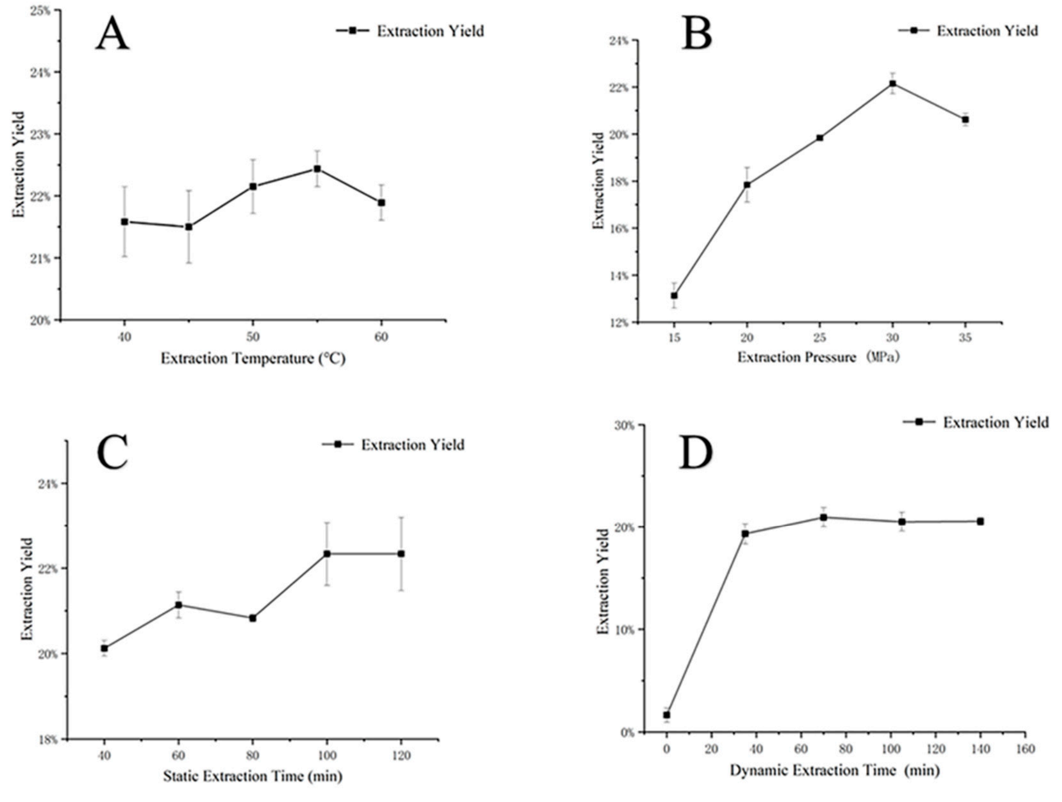

Fig. S1 Single factor analysis of SC-CO_2 extraction of PFSO: (A) The effect of extraction temperature on the extraction yield; (B) The effect of extraction pressure on the extraction yield; (C) The effect of static extraction time on the extraction yield; (D) The effect of dynamic extraction time on the extraction yield.

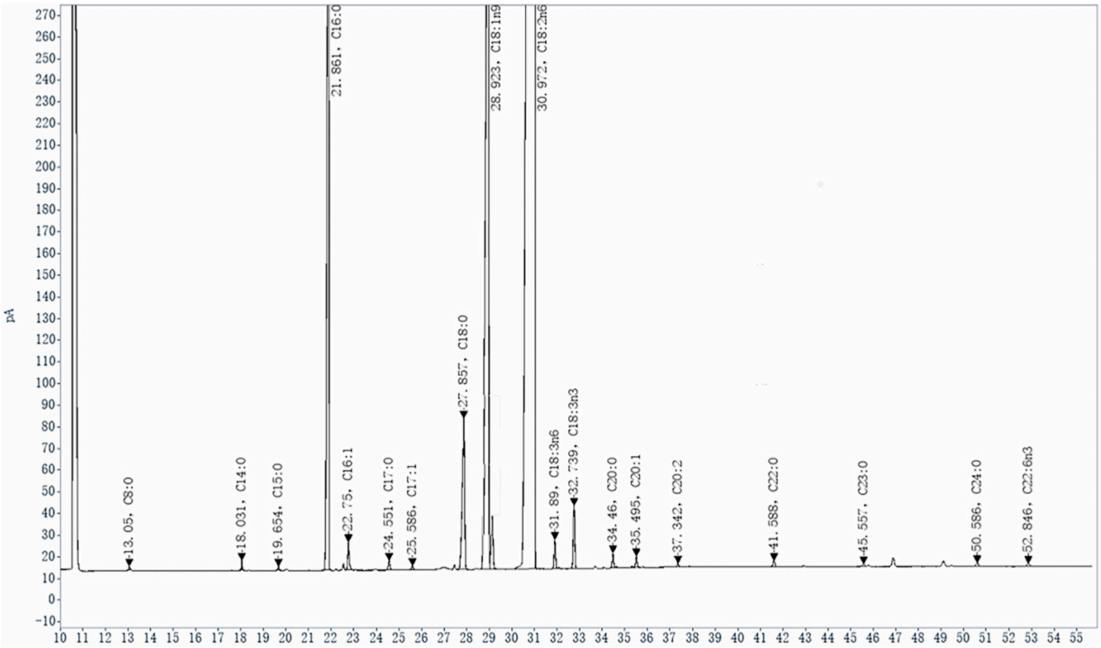

Fig. S2 GC chromatograms of PFSO.

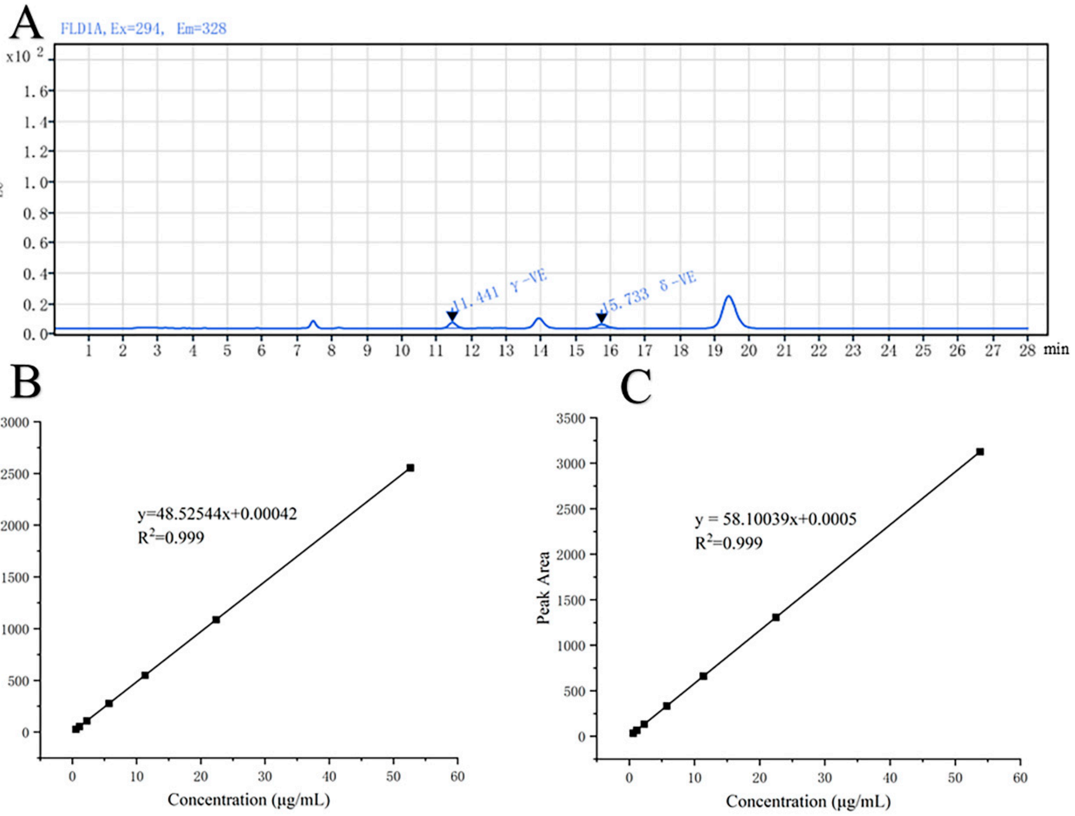

Fig. S3 Standard curves and liquid chromatograms of Ve in PFSO: (A) Liquid chromatograms of different concentrations of Ve standards; (B) Standard curve for γ-tocopherol; (C) Standard curve for δ-tocopherol

Table

Table S1 SC-CO_2 orthogonal array design

Factor	（A） Extraction temperature(℃)	（B） Extraction pressure (MPa)	（C） Dynamic extraction time (min)	（D） Static extraction time (min)
1	50	25	35	80
2	55	30	70	100
3	60	35	105	120

Number	（A）Extraction temperature (℃)	（B）Extraction pressure (MPa)	（C）Dynamic extraction time (min)	（D） Static extraction time (min)	Extraction yield (%)		
1	1	1	1	1	18.74	18.13	18.43
2	1	2	3	2	23.40	24.36	23.87
3	1	3	2	3	20.32	20.80	20.58
4	2	1	3	3	22.24	19.78	21.79
5	2	2	2	1	24.30	24.97	24.85
6	2	3	1	2	21.00	21.05	20.68
7	3	1	2	2	22.29	22.29	22.07
8	3	2	1	3	21.34	21.13	21.27
9	3	3	3	1	24.09	23.66	23.83
K_1	188.62	185.76	181.77	201.00			
K_2	200.66	209.49	202.47	201.01			
K_3	201.97	196.01	207.02	189.25			
k_1	20.96	20.64	20.20	22.33			
k_2	22.30	23.28	22.50	22.33			
k_3	22.44	21.78	23.00	21.03			
R	1.48	2.64	2.81	1.31			
	C>B>A>D	$A_3B_2C_3D_2$					

Table S2 GC-MS Analysis of PFSO

Number	Compound	Peak Area/%
1	Palmitic acid	3.91
2	Phthalic acid, bis-(10-hydroxy-decyl ester	1.63
3	Hexadecanoic acid, ethyl ester	0.67
4	7-Methyl-Z-tetradecen-1-ol acetate	0.60
5	Linoleic acid	51.53
6	Linoleic acid ethyl ester	3.88
7	Oleic acid	0.79
8	12-Methyl-E,E-2,13-octadecadien-1-ol	2.35
9	2-Hexadecanol	0.62
10	9,12-Octadecadienoic acid (Z,Z)-, 2-hydroxy-1-(hydroxymethyl)ethyl ester	5.81
11	Oleic acid, 3-(octadecyloxy)propyl ester	2.55
12	8-Octadecenoic acid, (2-phenyl-1,3-dioxolan-4-yl)methyl ester	2.57
13	Ethyl iso-allocholate	1.39
14	8,14-Seco-3,19-epoxyandrostane-8,14-dione, 17-acetoxy-3β-methoxy-4,4-dimethyl	0.53
15	Spirost-8-en-11-one, 3-hydroxy-, (3β,5α,14β,20β,22β,25R)	0.82
16	2,2,4-Trimethyl-3-(3,8,12,16-tetramethyl-heptadeca-3,7,11,15-tetra enyl)-cyclohexanol	13.89
17	β-Sitosterol	5.61

Table S3 Molecular docking results of tyrosinase with linoleic acid and β-Sitosterol.

Docked conformations	Amino acid residues	Binding forces
Tyrosinase-linoleic acid	LYS180	Hydrogen bond
complex	GLN41	Hydrophobic interaction
	PRO175	Hydrophobic interaction
	HIS178	Hydrophobic interaction
Tyrosinase-β-Sitosterol	TYR78	Hydrogen bond
complex	HIS76	Hydrophobic interaction
	TYR62	Hydrophobic interaction
	TYR98	Hydrophobic interaction
	PRO338	Hydrophobic interaction
	PRO349	Hydrophobic interaction
	GLU377	Hydrophobic interaction
